# TRIM5α Promotes Ubiquitination of Rta from Epstein–Barr Virus to Attenuate Lytic Progression

**DOI:** 10.3389/fmicb.2016.02129

**Published:** 2017-01-05

**Authors:** Hsiang-Hung Huang, Chien-Sin Chen, Wen-Hung Wang, Shih-Wei Hsu, Hsiao-Han Tsai, Shih-Tung Liu, Li-Kwan Chang

**Affiliations:** ^1^Department of Biochemical Science and Technology, College of Life Science, National Taiwan UniversityTaipei, Taiwan; ^2^Department of Internal Medicine, Kaohsiung Medical University HospitalKaohsiung, Taiwan; ^3^Molecular Genetics Laboratory, Department of Microbiology and Immunology, Chang-Gung UniversityTaoyuan, Taiwan

**Keywords:** TRIM5α, Rta, ubiquitination, Epstein–Barr virus, viral–host interactions

## Abstract

Replication and transcription activator (Rta), a key protein expressed by Epstein–Barr virus (EBV) during the immediate-early stage of the lytic cycle, is responsible for the activation of viral lytic genes. In this study, GST-pulldown and coimmunoprecipitation assays showed that Rta interacts *in vitro* and *in vivo* with TRIM5α, a host factor known to be involved in the restriction of retroviral infections. Confocal microscopy results revealed that Rta colocalizes with TRIM5α in the nucleus during lytic progression. The interaction involves 190 amino acids in the N-terminal of Rta and the RING domain in TRIM5α, and it was further found that TRIM5α acts as an E3 ubiquitin ligase to promote Rta ubiquitination. Overexpression of TRIM5α reduced the transactivating capabilities of Rta, while reducing TRIM5α expression enhanced EBV lytic protein expression and DNA replication. Taken together, these results point to a critical role for TRIM5α in attenuating EBV lytic progression through the targeting of Rta for ubiquitination, and suggest that the restrictive capabilities of TRIM5α may go beyond retroviral infections.

## Introduction

Epstein–Barr virus (EBV), a member of the human herpesvirus family, is an oncogenic virus that infects lymphoid and epithelial cells. Although EBV normally remains latent after infecting B lymphocyte cells, the virus must enter the lytic cycle to proliferate and produce infectious particles. The pivotal role of the two EBV intermediate-early proteins, Replication and transcription activator (Rta) and Zta, in activating the transcription of EBV lytic genes is well-documented (Speck et al., 1997; Zalani et al., 1997; Liu and Speck, 2003; Amon and Farrell, 2005). In previous studies, we showed that Rta is conjugated to SUMO-1 by Ubc9 and PIAS (protein inhibitor of activated STAT) proteins, and this sumoylation enhances Rta transactivation activity (Chang et al., 2004). Rta is also modified by SUMO-2 (Heilmann et al., 2010), and can be ubiquitinated via the SUMO chain by a SUMO-targeted E3 ubiquitin ligase, RNF4, thereby inhibiting EBV lytic activation (Yang et al., 2013).

In this study, we identified a second E3 ubiquitin ligase that promotes Rta ubiquitination to influence EBV lytic progression, TRIM5α. TRIM5α is a member of the tripartite motif (TRIM) protein superfamily, and acts as a host restriction factor that limits retroviral infection (Stremlau et al., 2004; Pertel et al., 2011). Earlier studies have shown that TRIM5α from rhesus macaques (rhTRIM5α) restricts early events in human immunodeficiency virus (HIV)-1 infection, while human TRIM5α restricts infection by N-tropic murine leukemia virus (N-MLV) (Yap et al., 2004). As an intrinsic immunity protein, TRIM5α catalyzes the synthesis of unanchored K63-linked polyubiquitin chains, and then activates NF-κB or AP-1 dependent transcription (Pertel et al., 2011). TRIM5α contains B boxes, a coiled-coil domain, and a RING domain, and can function as an E3 ubiquitin ligase (Reymond et al., 2001). In addition, TRIM5α contains a SPRY/B30.2 domain with two SUMO-interaction motifs (SIMs), which are required for N-MLV restriction (Arriagada et al., 2011). This SPRY/B30.2 domain can also directly bind with the HIV capsid, and is believed to be critical for HIV restriction (Sawyer et al., 2005; Stremlau et al., 2005; Luban, 2007; Ganser-Pornillos et al., 2011). Moreover, TRIM5α-mediated ubiquitin conjugation is required for HIV-1 capsid destabilization and inhibition of reverse transcription (Campbell et al., 2016). Interestingly, SIM mutations in TRIM5α not only lead to loss of restriction capability against N-MLV (Arriagada et al., 2011), but the mutations also prevent TRIM5α from shuttling into the cell nucleus, thus rendering it unable to restrict incoming HIV retrovirion cores (Brandariz-Nunez et al., 2013).

Although TRIM5α primarily acts to limit the propagation of retroviruses (Hatziioannou et al., 2004; Keckesova et al., 2004; Perron et al., 2004; Stremlau et al., 2004; Yap et al., 2004), it has been reported that rhTRIM5α can hamper the replication of herpes simplex virus (HSV) (Reszka et al., 2010), a double-stranded DNA virus. In this study, we identified an interaction between TRIM5α and the EBV viral protein, Rta, using GST-pulldown and coimmunoprecipitation assays. TRIM5α and Rta colocalized in the nucleus, and we further found that TRIM5α can act as an E3 ubiquitin ligase that promotes Rta ubiquitination. This subsequently leads to the downregulation of Rta transactivation capabilities, indicating that TRIM5α may play a critical role in attenuating EBV lytic progression. These results suggest that the restrictive abilities of TRIM5α are not limited to retroviruses, and may have interesting implications for antiviral research and development.

## Materials and Methods

### Cell Lines and EBV Lytic Induction

P3HR1, a Burkitt’s lymphoma cell line latently infected by EBV (Ben-Bassat et al., 1976), was cultured in RPMI1640 medium containing 10% fetal calf serum. 293T cells were cultured in Dulbecco’s modified Eagle’s medium (DMEM) containing 10% fetal calf serum. P3HR1 cells were treated with 30 ng/mL 12-*O*-tetradecanoylphorbol-13-acetate (TPA) and 3 mM sodium butyrate to activate the EBV lytic cycle (Luka et al., 1979; Davies et al., 1991; Chang and Liu, 2000).

### Plasmids

Plasmids pGEX-TRIM5α and pEGFP-TRIM5α were constructed by inserting a DNA fragment, which was amplified by PCR using pLPCX-TRIM5α as a template (NIH AIDS reagent program, USA) and the primers TRIM5α-F (5′-CCGGAATTCATGGCTTCTGGAATCCTGGT) and TRIM5α-R (5′-ACGCGTCGACTCAAGAGCTTGGTGAGCACA), into the *Eco*RI and *Sal*I sites in pGEX-4T1 (Amersham) and pEGFP-C2 (Clontech), respectively. DNA fragments, which encode the regions in TRIM5α from amino acids 1 to 261, 81 to 493, and 261 to 493 were amplified by PCR. These DNA fragments were then inserted into the *Eco*RI and *Sal*I sites in pEGFP-C2 to generate pEGFP-TRIM5α-dC, pEGFP-TRIM5α-dN, and pEGFP-TRIM5α-dNM, respectively. The DNA fragment from amino acids 1 to 261 was also inserted into pTag2B to create pFlag-TRIM5α-dC. Plasmids that express deletion mutants of GFP-Rta, including GFP-N190, GFP-N191/415, and GFP-Rev have been described previously (Hsu et al., 2005; Chang et al., 2010; Yang and Chang, 2013). Plasmids pFlag-Ub, pFlag-Rta, and pET-Rta, which express Flag-tagged ubiquitin, Flag-tagged Rta and His-tagged Rta, were described earlier (Yang et al., 2013). For transient transfection assays, the reporter plasmid, pBMRF1, was constructed by inserting a PCR-amplified DNA fragment containing the -172 to +20 region in BMRF1 into pGL2-Basic (Chang et al., 2010). Plasmid pBMLF1-RRE containing the RRE sequence from the BMLF1 promoter and a TATA sequence was synthesized and inserted into pGL2-Basic (Chang et al., 2004). Similarly, the RRE sequence from the BMRF1 promoter and a TATA sequence was synthesized and inserted into pGL2-Basic to generate pBMRF1-RRE.

### MALDI-TOF Mass Spectrometry

P3HR1 cells were treated with TPA and sodium butyrate for 24 h to activate the EBV lytic cycle and Rta expression. Cells were harvested by low speed centrifugation and lysed using mRIPA buffer (50 mM Tris-HCl (pH 7.8), 150 mM NaCl, 5 mM EDTA, 0.5% Triton X-100, 0.5% Nonidet P-40), and proteins in the lysate were immunoprecipitated using anti-Rta antibody and protein A/G-agarose beads (Millipore). The beads were washed with mRIPA buffer for three times, and proteins on the beads were then extracted with electrophoresis sample buffer (10% glycerol, 60 mM Tris-HCl pH 6.8, 2% SDS, 2.5% β-mercaptoethanol, 2 mM EDTA) and subjected to 2-D polyacrylamide gel electrophoresis. Proteins in the gel were stained with Coomassie blue, and prospective protein spots in the gel were then excised. The proteins in the spots were digested with trypsin according to an in-gel digestion protocol (Shevchenko et al., 1996), and the resulting peptides were analyzed using a Bruker Biflex III MALDI-TOF mass spectrometer (Bruker Daltonics) (Wu et al., 2007). The m/z ratios of the digested peptides and their fragmented ions were used to search the annotated human genome in the Mass Spectrometry protein sequence Database (MSDB), using Mascot search software v1.8 (Matrix Science Inc). The search criteria used were as follows: maximum of one missed trypsin cleavage; variable modification, including carbamidomethylation; and 1 Da peptide mass tolerance. Only proteins identified as significant hits (*p* < 0.05) by Mascot peptide mass fingerprint search were selected.

### Protein Expression and GST Pulldown Assay

*Escherichia coli* BL21(DE3)(pGEX-TRIM5α) and *E. coli* BL21(DE3)(pGST) were cultured to the mid-log phase and then treated with 0.1 mM isopropyl β-D-1-thiogalactopyranoside (IPTG) to, respectively, induce the expression of GST-TRIM5α and GST according to a method described earlier (Chang et al., 2004). GST and GST-TRIM5α were purified from bacterial lysates using glutathione-Sepharose 4B beads (GE healthcare).

### Transient Transfection and Luciferase Assay

293T cells were transfected with plasmids using Turbofect (Thermo Fisher Scientific), according to the method recommended by the manufacturer. At 24–48 h after transfection, cells were harvested and lysed using mRIPA lysis buffer [50 mM Tris-HCl (pH 7.8), 150 mM NaCl, 5 mM EDTA, 0.5% Triton X-100, 0.5% Nonidet P-40]. Luciferase assays were performed according to a method described earlier (Chang et al., 1998).

### Coimmunoprecipitation of Rta and TRIM5α

293T cells were transfected with pCMV-Rta and pHA-TRIM5α, and at 24 h after transfection, cells were collected and lysed in mRIPA buffer. Proteins in the lysate were immunoprecipitated with anti-Rta and anti-HA antibodies. Protein A/G-agarose beads were then added to the lysate, and proteins bound to the beads were subsequently analyzed by immunoblotting. To detect ubiquitinated proteins, 293T cells were cotransfected with pCMV-R, pTag-2B, and pLPCX-TRIM5α. At 24 h after transfection, cells were treated with 5 μM MG132 for additional 12 h. Cells were harvested according to a method described earlier (Chang et al., 2004; Yang et al., 2013) to detect ubiquitin-conjugated proteins.

### Immunoblot Analysis

Proteins were separated in SDS-polyacrylamide gels and then electrotransferred to Hybond C membranes (GE) at 90 V for 1 h, according to a method described elsewhere (Chang et al., 2004). The membrane was then probed with the appropriate antibodies, including anti-Rta (Argene), anti-TRIM5α (Santa Cruz), anti-HA (Roche), anti-GFP (Santa Cruz), anti-GST (Santa Cruz), anti-VCA (Argene), anti-BFRF3 (Wang et al., 2015), and anti-α-tubulin (Sigma) antibodies.

### Immunofluorescence Analysis

P3HR1 cells were treated with sodium butyrate and TPA for 24 h, harvested by centrifugation, plated on poly-L-lysine (Sigma)-coated coverslips, and fixed with 4% paraformaldehyde in PBS for 30 min. Immunostaining was conducted using anti-Rta monoclonal antibodies (Argene) and anti-TRIM5α polyclonal antibodies (Santa Cruz). Cells were then treated with Alexa Fluor^®^ 594 goat anti-mouse and Alexa Fluor^®^ 488 goat anti-rabbit antibodies (Invitrogen). Nuclei were visualized by staining with 5 mg/mL 4′-6-diamidino-2-phenylindole (DAPI). Cells were observed under a confocal laser scanning microscope (Leica TCS SP8).

### Knockdown of TRIM5α Expression

TRIM5α shRNA and plasmids, including pMD2.G, pCMVDR8.91, and pLKO-shRNA, were purchased from the National RNAi Core Facility, Genomic Research Center, Academia Sinica, Taipei, Taiwan. 293T cells (2 × 10^5^) were cotransfected with plasmids expressing TRIM5α shRNA (target sequence: 5′-CCAGACATTTGTGAATTTCAA-3′; 2.25 μg), helper plasmids pMD2.G (0.25 μg) and pCMVDR8.91 (2.25 μg), using Turbofect *in vitro* transfection reagent (Thermo Fisher Scientific). Culture media was changed on the following day, and after an additional 24 h, viral supernatants were collected and filtered (0.22 μM), then stored at -80°C. Plasmid pLKO-shRNA was used as a negative control. For lentivirus infection, P3HR1 cells (3 × 10^5^/mL) were transduced with the generated lentiviruses, together with 5 μg/mL of polybrene. Infected P3HR1 cells were selected using 2 μg/mL puromycin in culture medium to produce stable cell lines according to the protocol^1^.

### Determining Copy Numbers of EBV DNA

P3HR1 cells were treated with TPA and sodium butyrate to induce the lytic cycle. After 5 days of culturing, virus particles released into the medium were collected by ultracentrifugation at 25,000 × *g* for 2.5 h. EBV copy numbers were determined according to a method described previously (Ryan et al., 2004; Chiu et al., 2007). A standard curve was established using maxi-EBV DNA purified from *E. coli* after qPCR analysis. The amounts of EBV DNA purified from the virus in the culture medium were similarly analyzed and compared with the standard curve.

## Results

### Identification of Cellular Proteins Interacting with Rta

**FIGURE 1:**
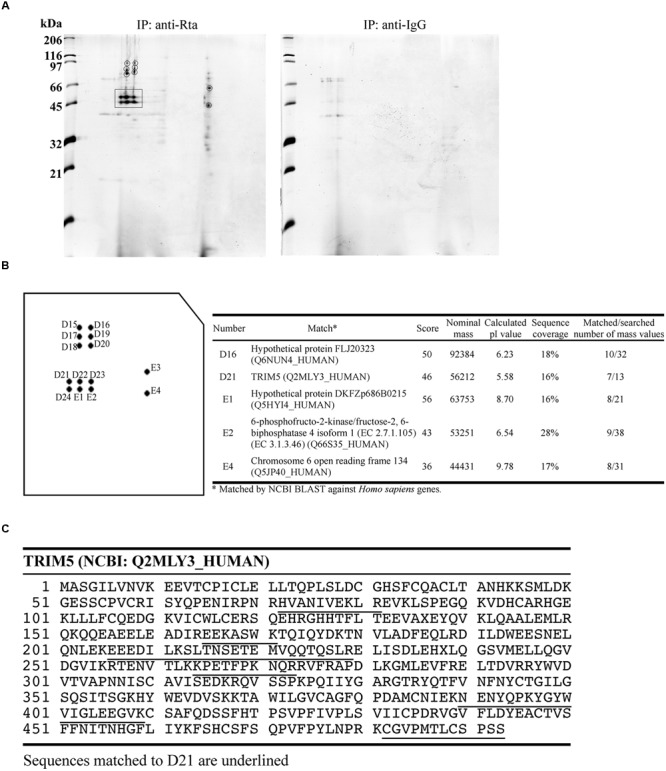
**MALDI-TOF mass spectrometry analysis of proteins that interact with Rta. (A)** P3HR1 cells were treated with TPA and sodium butyrate for 24 h to induce the EBV lytic cycle and Rta expression. Cells were then lysed, and proteins in the lysate were immunoprecipitated (IP) with anti-Rta or anti-IgG antibodies. Immunoprecipitated proteins were denatured and analyzed by 2-D polyacrylamide gel electrophoresis. **(B)** Proteins in prospective spots were digested with trypsin and analyzed by MALDI-TOF mass spectrometry. Proteins with peptide fingerprints matching those in the MSDB database are listed in the table at right. **(C)** Peptide sequences in TRIM5α matching those in the D21 proteins are underlined.

We treated P3HR1 cells with TPA and sodium butyrate for 24 h to activate the EBV lytic cycle and allowed the expression of Rta. Proteins interacting with Rta were then coimmunoprecipitated from cell lysates, using anti-Rta antibodies. A similar immunoprecipitation experiment was conducted using anti-IgG antibody as a control. Afterward, 2-D polyacrylamide gel electrophoresis of the immunoprecipitated proteins from each experiment (**Figure 1A**) was conducted, and the gels were then stained with Coomassie blue and compared. The comparison results revealed 14 protein spots that appeared in the gel containing anti-Rta immunoprecipitated proteins, but not in the control gel (**Figure 1A**). After digesting the proteins in these spots with trypsin, the resulting peptides were analyzed by MALDI-TOF mass spectrometry. The results revealed that five protein spots had peptide fingerprints matching those in the MSDB database (**Figure 1B**). Among these, Spot D21 had a protein with a fingerprint matching that of TRIM5α (**Figure 1C**), a known E3 ubiquitin ligase that promotes protein ubiquitination. Since Rta is known to be modified by ubiquitin (Yang et al., 2013), this study further sought to investigate whether TRIM5α influences the ubiquitination status and functions of Rta.

### Rta Interacts with TRIM5α *In vitro* and *In vivo*

**FIGURE 2:**
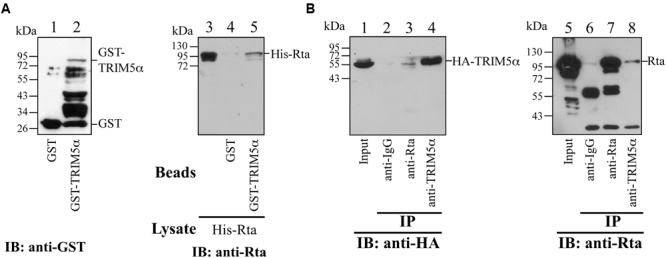
**Interaction between Rta and TRIM5α. (A)** In a GST-pulldown assay, His-Rta (lanes 3–5) was mixed with GST- (lane 4) and GST-TRIM5α- (lane 5) glutathione-Sepharose beads. Proteins pulled down by the beads were analyzed by immunoblotting (IB) with anti-Rta antibody. Proteins on the glutathione-Sepharose beads (lanes 1 and 2) and Rta in 1% of the lysate (lane 5) were also detected by immunoblotting. **(B)** Coimmunoprecipitation assay results. Anti-Rta and anti-TRIM5α antibodies were added to the lysate from 293T cells that had been transfected with pLPCX-HA-TRIM5α and pCMV-Rta. Lanes 1 and 5 were loaded with 3% of the cell lysate. Proteins immunoprecipitated (IP) with anti-Rta and anti-TRIM5α antibodies or anti-IgG antibody were detected by immunoblotting (IB), using anti-HA (lanes 1–4) and anti-Rta antibodies (lanes 5–8).

To verify the interaction between Rta and TRIM5α, we expressed GST-TRIM5α and GST in *E. coli*, and bound these proteins to glutathione-Sepharose beads. GST-TRIM5α-glutathione-Sepharose beads were then added to *E. coli* BL21(DE3)(pET-Rta) lysates, and proteins pulled down by the beads were detected by immunoblotting with anti-Rta antibody. The results revealed that GST-TRIM5α, but not GST-glutathione-Sepharose beads, pulled down Rta (**Figure 2A**, lanes 4 and 5), providing *in vitro* evidence of a direct interaction between TRIM5α and Rta. A coimmunoprecipitation experiment using lysates from 293T cells that had been cotransfected with pCMV-Rta and pLPCX-HA-TRIM5α similarly showed that anti-TRIM5α antibody immunoprecipitated HA-TRIM5α and coimmunoprecipitated Rta (**Figure 2B**, lanes 4 and 8), while anti-Rta antibody immunoprecipitated Rta and coimmunoprecipitated HA-TRIM5α (**Figure 2B**, lanes 3 and 7).

### Colocalization of TRIM5α with Rta in the Nucleus

**FIGURE 3:**
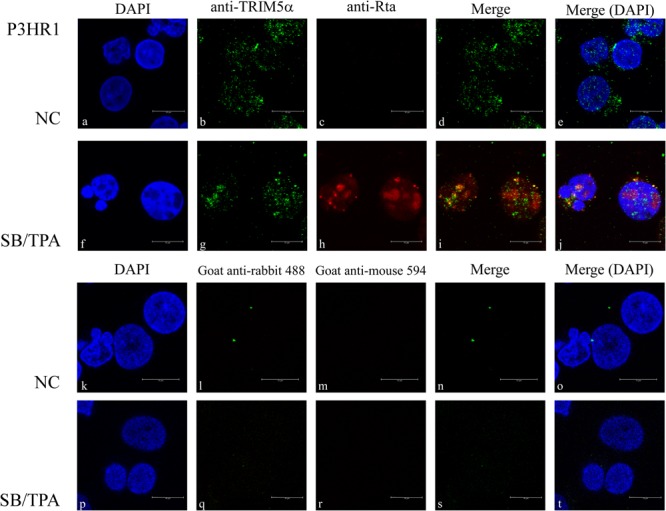
**Subcellular localization of Rta and TRIM5α.** P3HR1 cells were treated with sodium butyrate and TPA (SB/TPA) or DMSO (NC) for 24 h. Cells were incubated with monoclonal anti-Rta antibody **(c,h)** and polyclonal anti-TRIM5α antibody **(b,g)**. Cells were also stained with secondary antibodies **(k–t)** as a control. Nuclei were visualized by DAPI staining **(a,f)**. Cells were observed under a confocal laser-scanning microscope, and **(d,e,i,j,n,o,s,t)** are merged images. Scale bar indicates 10 μm.

The localization of Rta and TRIM5α in P3HR1 cells treated with TPA and sodium butyrate for 24 h was examined by indirect immunofluorescence. Under a confocal laser scanning microscope, we found that both Rta and TRIM5α formed speckles, many of which colocalized in the cell nucleus (**Figures 3f–j**). However, Rta was not observed if the cells were not treated with TPA and sodium butyrate (**Figures 3a–e**). P3HR1 cells treated with or without TPA and sodium butyrate were also stained with secondary antibodies as a control to demonstrate the specificity of anti-TRIM5α antibody (**Figures 3k–t**).

### Analysis of Interacting Domains in Rta and TRIM5α

**FIGURE 4:**
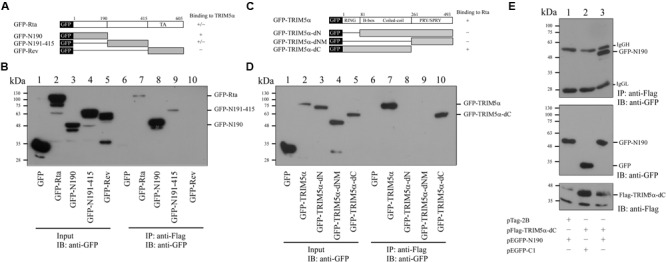
**Mapping the interaction domains in Rta and TRIM5α. (A)** Plasmids that expressed GFP fused to different segments of Rta were used to delineate the region in Rta that interacts with TRIM5α. **(B)** 293T cells were cotransfected with pFlag-TRIM5α and plasmids expressing GFP or GFP-Rta fusion proteins (pEGFP-Rta, pEGFP-N190, pEGFP-N191-415, pEGFP-Rev, or pEGFP-C1). Input lanes were loaded with 5% of the lysate (lanes 1–5). Proteins in the lysates were coimmunoprecipitated (IP) with anti-Flag antibody and analyzed by immunoblotting (IB) using anti-GFP antibody (lanes 6–10). **(C)** Plasmids expressing various GFP-TRIM5α fusion proteins (GFP-TRIM5α, GFP-TRIM5α-dN, GFP-TRIM5α-dNM, GFP-TRIM5α-dC) were generated. **(D)** 293T cells were cotransfected with plasmids encoding GFP or GFP-TRIM5α fusion proteins (GFP-TRIM5α, GFP-TRIM5α-dN, GFP-TRIM5α-dNM, GFP-TRIM5α-dC). Input lanes were loaded with 5% of the lysate, and GFP-fusion protein expression levels were detected using anti-GFP antibody (lanes 1–5). Proteins in the lysates were coimmunoprecipitated with anti-Flag antibody and analyzed by immunoblotting using anti-GFP antibody (lanes 6–10). **(E)** 293T cells were cotransfected with pFlag-TRIM5α-dC and plasmids expressing either GFP-N190 or GFP. Input lanes were loaded with 5% of the lysate. Proteins in the lysates were coimmunoprecipitated with anti-Flag antibody and analyzed by immunoblotting using anti-GFP antibody.

We cotransfected 293T cells with pFlag-TRIM5α and plasmids expressing different segments of Rta that were fused to GFP (**Figure 4A**). Control cells were cotransfected with plasmids expressing GFP and Flag-TRIM5α. Immunoblotting with anti-GFP antibody revealed that GFP and the GFP-Rta fusion proteins were expressed at similar levels after transfection (**Figure 4B**, lanes 1–5). Following immunoprecipitation with anti-Flag antibody, precipitated proteins were analyzed by immunoblotting with anti-GFP antibody. Results showed that GFP-N190 (**Figure 4B**, lane 8), which contains 190 amino acids from the N-terminal of Rta, was coimmunoprecipitated with Flag-TRIM5α, indicating that this is the region in Rta that interacts with TRIM5α. In addition, we also observed a weak binding effect between TRIM5α and GFP-N191-415 (**Figure 4B**, lane 9). However, GFP-Rev was not coimmunoprecipitated by Flag-TRIM5α (**Figure 4B**, lane 10). A control experiment showed that Flag-TRIM5α was not coimmunoprecipitated with GFP (**Figure 4B**, lane 6). These results were reproduced from at least three independent experiments. To identify the region in TRIM5α that interacts with Rta, we generated plasmids expressing GFP fused to different segments of TRIM5α (**Figure 4C**). Following cotransfection of these plasmids with pFlag-Rta to 293T cells, cells were subsequently lysed, and the lysates subjected to immunoprecipitation using anti-Flag antibody (**Figure 4D**, lanes 1–5). Immunoblot analysis with anti-GFP antibody revealed that only GFP-TRIM5α and GFP-TRIM5α-dC were coimmunoprecipitated with Flag-Rta (**Figure 4D**, lanes 7 and 10). In addition, 293T cells were cotransfected with plasmids encoding GFP-N190 and Flag-TRIM5α-dC, and immunoblot analysis revealed that GFP-N190 in cell lysates (**Figure 4E**, lane 3) was immunoprecipitated by anti-Flag antibody (**Figure 4E**, lane 3). However, control cells that were cotransfected with plasmids expressing GFP and Flag-TRIM5α-dC revealed that GFP was not coimmunoprecipitated with Flag-TRIM5α-dC (**Figure 4E**, lane 2). These results indicate that the N-terminal of Rta interacts with the N-terminal RING domain in TRIM5α.

### TRIM5α Promotes Rta Ubiquitination

**FIGURE 5:**
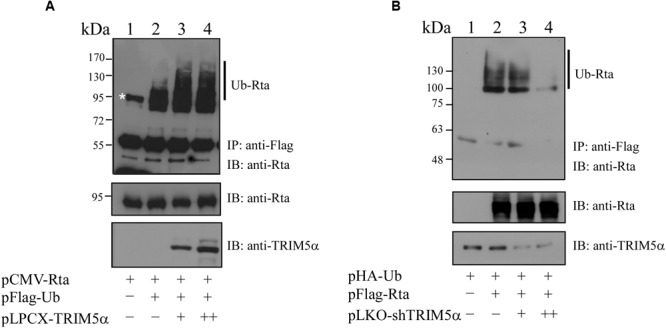
**Enhancement of Rta ubiquitination by TRIM5α. (A)** 293T cells were cotransfected with plasmids expressing Rta, Flag-ubiquitin, and TRIM5α. Proteins in the lysates were immunoprecipitated (IP) using anti-Flag antibody and assessed by immunoblotting (IB) with anti-Rta antibody. **(B)** 293T cells were cotransfected with plasmids pFlag-Rta, pHA-Ub, and pLKO-shTRIM5α, which encoded Flag-Rta, HA-ubiquitin and shTRIM5α, respectively. Proteins in the lysates were immunoprecipitated (IP) using anti-Flag antibody and analyzed by immunoblotting (IB) with anti-HA antibody. At 24 h after transfection, cells were treated with 5 μM MG132 for additional 12 h to inhibit proteasome degradation. The asterisk indicates a non-specific band, also detected in previous studies (Chang et al., 2004; Yang et al., 2013). Ub-Rta, ubiquitinated Rta.

TRIM5α has previously been shown to be an E3 ubiquitin ligase (Yamauchi et al., 2008), and the observation of Rta-TRIM5α interaction prompted us to investigate whether TRIM5α can influence Rta ubiquitination. We proceeded to transfect 293T cells with pCMV-Rta alone, or with pCMV-Rta and pFlag-Ub. At 24 h after transfection, cells were treated with MG132, a proteasome inhibitor, to prevent the degradation of Rta. When proteins from the lysates of cells transfected with pCMV-Rta alone were immunoprecipitated using anti-Flag antibody and assessed by immunoblotting with anti-Rta, a single non-specific band of about 95 kDa was detected (**Figure 5A**, lane 1), which was also observed in previous studies (Chang et al., 2004; Yang et al., 2013). In 293T cells cotransfected with pCMV-Rta and pFlag-Ub, low amounts of ubiquitinated Rta were detected after the proteins in the lysates were immunoprecipitated with anti-Flag antibody and assessed by immunoblotting with anti-Rta antibody (**Figure 5A**, lane 2). When cells were cotransfected with pCMV-Rta, pFlag-Ub, and 0.3 or 0.6 μg of pLCPX-TRIM5α, ubiquitinated Rta became more prominent (**Figure 5A**, lanes 3 and 4), demonstrating that TRIM5α promotes Rta ubiquitination. Subsequently, 293T cells were transfected with TRIM5α shRNA to determine whether this would reduce Rta ubiquitination. In a control experiment, immunoblotting did not detect ubiquitinated Rta in cells that were transfected with pHA-Ub (**Figure 5B**, lane 1). Ubiquitinated Rta was also detected in cells that were cotransfected with pFlag-Rta and pHA-Ub (**Figure 5B**, lane 2). However, introducing TRIM5α shRNA reduced the amounts of ubiquitinated Rta (**Figure 5B**, lanes 3 and 4).

### Influence of TRIM5α on Rta Transactivation Activity

**FIGURE 6:**
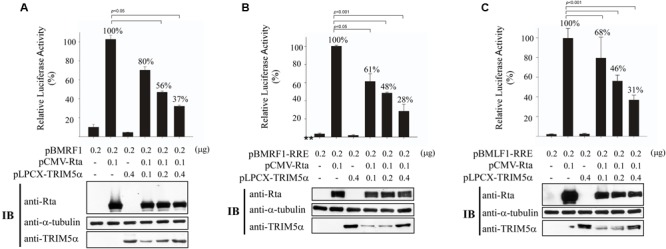
**Reduction of Rta transactivation activity by TRIM5α.** 293T cells were cotransfected with pCMV-R, pLPCX-TRIM5α, and **(A)** pBMRF1, **(B)** pBMRF1-RRE, or **(C)** pBMLF1-RRE. Luciferase activities were assessed 24 h after transfection. Each transfection experiment was performed three times, and each sample in the experiment was prepared in duplicate. Rta, TRIM5α, and α-tubulin expressed by the cells were examined by immunoblotting (IB). The data obtained from the reporter assay were subjected to one-way analysis of variance (ANOVA) using SPSS software 12.0. *P*-values of <0.05 were considered statistically significant. RLU, relative light units.

It is known that Rta acts as a key immediate-early protein that transactivates viral lytic genes to move EBV into the lytic cycle. Therefore, we sought to evaluate the impact of enhanced TRIM5α expression and Rta ubiquitination on the transactivation capabilities of Rta. In a transient transfection assay, we examined how TRIM5α expression can influence Rta transactivation of the EBV BMRF1 promoter, using a luciferase reporter plasmid, pBMRF1 (Chang et al., 2004). After cotransfecting 293T cells with pCMV-Rta and pBMRF1, Rta transactivation of BMRF1 was measured by luciferase activity, and the values were taken as 100% (**Figure 6A**). We further included 0.1–0.4 μg of pLPCX-TRIM5α in cotransfections, and found that enhanced expression of TRIM5α gradually reduced BMRF1 promoter activation in a dose-dependent manner to just 37–80% (**Figure 6A**). A similar experiment also showed that cotransfection of pLPCX-TRIM5α similarly disrupted the ability of Rta to transactivate the BMRF1-RRE (**Figure 6B**) and BMLF1-RRE (**Figure 6C**) promoters, which contain Rta-responsive elements (RREs). These results show that overexpression of TRIM5α decreases Rta transactivation capability.

### Influence of TRIM5α on the Expression of EBV Lytic Proteins and Virion Production

**FIGURE 7:**
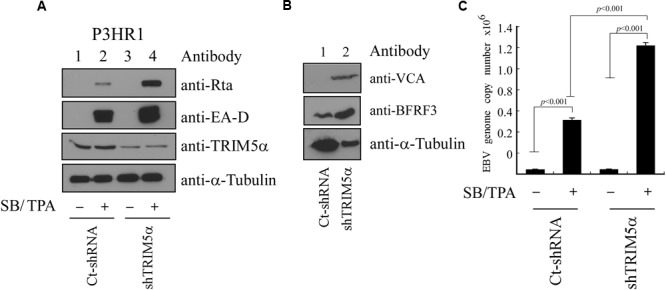
**Influence of TRIM5α on EBV lytic progression. (A)** P3HR1 cells were infected by lentivirus expressing TRIM5α shRNA (shTRIM5α) or control shRNA (Ct-shRNA). Thereafter, cells were treated with sodium butyrate and TPA (SB/TPA) for 48 h, and proteins in the lysates were examined by immunoblotting with anti-Rta, anti-EA-D, anti-TRIM5α, and anti-α-tubulin antibodies. **(B)** Cells were lysed at 120 h after TPA and sodium butyrate treatment, and the expression of VCA, BFRF3, and α-tubulin was examined by immunoblotting. **(C)** An EBV lytic DNA replication assay was conducted by qPCR to determine the copy numbers of EBV DNA. The amount of EBV DNA was determined by standard curve, which was in turn established using maxi-EBV DNA purified from *E. coli* after qPCR analysis. The data were subjected to one-way analysis of variance (ANOVA) using SPSS software 12.0. *P*-values of <0.05 were considered statistically significant.

P3HR1 cells were infected with lentivirus expressing TRIM5α shRNA or control shRNA, and cells were then treated with TPA and sodium butyrate to activate the EBV lytic cycle. We found that, compared with cells infected with control shRNA, infection by lentivirus expressing TRIM5α shRNA caused cells to express less TRIM5α, but more Rta and EA-D (**Figure 7A**). The expression of TRIM5α shRNA also led to increases in the expression of two EBV capsid proteins, VCA and BFRF3 (**Figure 7B**). Quantitative PCR results showed that after lytic activation of P3HR1 cells infected with lentivirus expressing control shRNA, viral yield was estimated at 3 × 10^5^ EBV particles. However, for cells infected with lentivirus expressing TRIM5α shRNA, viral yields increased 400% to 1.2 × 10^6^ viral particles (**Figure 7C**). These results showed that TRIM5α expression serves to attenuate EBV lytic development.

## Discussion

Rta is a key immediate-early viral protein that is responsible for activating the transcription of EBV lytic genes, thereby triggering the viral lytic cascade (Liu and Speck, 2003; Amon and Farrell, 2005; Hsu et al., 2005). In this study, we utilized MALDI-TOF mass spectrometry to identify host proteins that can interact with Rta and affect Rta activation of the EBV lytic cycle. From the mass spectrometry results, we identified TRIM5α as an Rta-interacting protein (**Figure 1**). Considering that TRIM5α is a known E3 ubiquitin ligase (Yamauchi et al., 2008), while Rta ubiquitination has also been reported to affect its transactivation capabilities (Yang et al., 2013), we therefore sought to confirm whether TRIM5α influences Rta ubiquitination, and if so, whether Rta transactivation ability is affected as a result. Our results also suggest that TRIM5α may be abundantly present in P3HR1 cells, as levels of TRIM5α were detectable by Coomassie blue staining of a 2-D polyacrylamide gel. Our previous studies identified cellular Rta-interacting proteins, such as MCAF1 and ATF2 (Chang et al., 2005; Lin et al., 2014), which were not detected by MALDI-TOF mass spectrometry analysis. It is likely that the amounts of these proteins binding to Rta are less than that of TRIM5α, causing the proteins to escape detection. In order to validate the interaction between Rta and TRIM5α, we conducted a GST-pulldown assay, and showed that GST-TRIM5α-glutathione-Sepharose beads pulled down His-Rta (**Figure 2A**, lane 5) via the N-terminal region in Rta and the N-terminal RING domain in TRIM5α (**Figure 4**). Conversely, anti-Rta antibody was also shown to coimmunoprecipitate HA-TRIM5α (**Figure 2B**, lanes 3 and 8). Confocal microscopy results revealed that Rta colocalizes with TRIM5α in the nuclei of P3HR1 cells after EBV lytic induction (**Figure 3**). Taken together, these results corroborate the mass spectrometry findings, and provide supporting evidence for Rta-TRIM5α interaction. Interestingly, previous studies have shown that TRIM5α primarily resides in the cytoplasm, so as to defend against incoming retroviral virions (Stremlau et al., 2004). We also examined the subcellular localization of GFP-TRIM5α and TRIM5α in 293T cells, and found that TRIM5α formed dots in the cytoplasm (data not shown). However, our findings suggest that the majority of TRIM5α is present in the nuclei of P3HR1 cells, rather than the cytoplasm (**Figure 3**). Whether this is an anomaly that exists only in P3HR1 or lymphocyte cells, or a response to activation of the viral lytic cycle by previously latent EBV in the cell nuclei remains to be determined. It has been reported that TRIM5α can shuttle between the cytoplasm and nucleus in a manner that is dependent on amino acids 60–93 in the N-terminal of TRIM5α, although there is no significant influence to the antiviral activity (Diaz-Griffero et al., 2011). Still, the factors driving TRIM5α shuttling have not been identified as yet, and it is possible that TRIM5α may relocate in response to different viral insults, via mechanisms that remain to be elucidated.

TRIM5α is known to act as an E3 ubiquitin ligase, and here we found that TRIM5α can promote Rta ubiquitination (**Figure 5A**). Introducing TRIM5α shRNA inhibits the levels of ubiquitinated Rta in 293T cells (**Figure 5B**). However, we were unable to detect the difference in the amounts of ubiquitinated Rta before and after the knockdown of TRIM5α in P3HR1 cells. EBV is known to express at least three deubiquitinases, including BSLF1, BPLF1, and BXLF1 (Schlieker et al., 2005; Sompallae et al., 2008). This may stabilize Rta, making the detection of its ubiquitination in P3HR1 cells more difficult. We also found that USP11, a deubiquitinase that acts against RNF4 activity (Hendriks et al., 2015), removes the ubiquitin chains on Rta efficiently (Chen et. al., unpublished results), suggesting that the ubiquitination of Rta is tightly regulated under physiological conditions. Additionally, overexpression of TRIM5α appears to hamper Rta transactivation of the EBV lytic cycle, as intracellular amounts of Rta, EA-D, BFRF3, and VCA viral lytic proteins in P3HR1 cells treated with sodium butyrate and TPA decreased with TRIM5α overexpression; moreover, expression of TRIM5α reduced the number of EBV virions produced by P3HR1 cells (**Figure 7**). In a transient transfection study, we showed that the ability of Rta to transactivate three EBV lytic promoters is negatively affected in a dose-dependent manner by enhanced expression of TRIM5α (**Figure 6**). Furthermore, the expression of TRIM5α shRNA significantly increased the number of EBV viral particles produced by P3HR1 cells treated with sodium butyrate and TPA (**Figure 7**). These results show that TRIM5α expression attenuates Rta ability to activate the transcription of EBV lytic genes and promote EBV lytic development, and suggests that the antiviral properties of TRIM5α may extend beyond retroviruses.

So far, only a few proteins, including NK-κB and hTERT, are known to decrease the lytic potential of EBV (Cahir-McFarland et al., 2000; Terrin et al., 2007). Among these two proteins, NK-κB is known to inhibit EBV lytic transcription and replication (Brown et al., 2003), while hTERT disrupts the EBV lytic cycle via a mechanism that is as yet unclear (Terrin et al., 2007). Our previous research indicated that RNF4 can target SUMO-2-Rta to enhance the ubiquitination of Rta, thereby inhibiting EBV lytic progression (Yang et al., 2013). Here, we report another cellular protein, TRIM5α, which also can play a negative role in hampering EBV lytic development. It is known that Rta is constitutively expressed, particularly in epithelial cells (Zalani et al., 1992). The presence of TRIM5α may reduce Rta expression levels at this stage and allow the virus to be maintained in latency. Further research into the role of TRIM5α and its ability to influence EBV and other viral physiology may be warranted.

In summary, we demonstrate that an E3 ubiquitin ligase, TRIM5α, can interact in the cell nucleus with the EBV immediate-early protein, Rta. TRIM5α promotes Rta ubiquitination, and this subsequently disrupts Rta ability to transactivate EBV lytic genes. Overexpression of TRIM5α was found to reduce viral promoter activation and viral lytic gene expression in a dose-dependent manner. Moreover, TRIM5α reduced EBV virion production in P3HR1 cells treated with sodium butyrate and TPA to induce EBV lytic activation, while the application of TRIM5α shRNA significantly increased the production of EBV viral particles. Taken together, these results indicate that TRIM5α can hamper Rta transactivation via the promotion of Rta ubiquitination, and suggest that the antiviral properties of TRIM5α may not be limited to retroviruses.

## Author Contributions

C-SC, S-TL, and L-KC designed the study; H-HH, C-SC, W-HW, S-WH and H-HT conceived and performed the experiments; H-HH and C-SC conducted statistical analysis; H-HH, C-SC, S-TL and L-KC wrote the manuscript.

## Conflict of Interest Statement

The authors declare that the research was conducted in the absence of any commercial or financial relationships that could be construed as a potential conflict of interest. The reviewer DJH and handling Editor declared their shared affiliation, and the handling Editor states that the process nevertheless met the standards of a fair and objective review.

## Footnotes

1 http://rnai.genmed.sinica.edu.tw/webContent/web/protocols
